# The role of parasite manipulation in vector-borne diseases

**DOI:** 10.1093/emph/eoz019

**Published:** 2019-06-20

**Authors:** Meredith R Spence Beaulieu

**Affiliations:** Department of Entomology and Plant Pathology, North Carolina State University, Raleigh, NC, USA

## DEFINITION AND BACKGROUND

The parasite manipulation hypothesis posits that parasites can purposefully alter host behaviours, increasing probability of transmission to an uninfected host [1]. An example is *Toxoplasma gondii*, where infected rodents become less predator averse, increasing the likelihood of infection reaching the feline host [2]. With other behavioural alterations, determination of whether effects are due to manipulations or are secondary outcomes of infection can be difficult [1]. Regardless, parasite-induced changes represented in the manipulation hypothesis have implications for disease transmission.

The hypothesis applies to vector-borne diseases, where parasite-induced changes in vector behaviour can increase transmission to the non-arthropod host. Here, a commonly affected behaviour is blood-feeding. Arthropods must blood-feed twice to transmit pathogens, first on an infectious host then again on a susceptible host. This necessity for two blood meals to fulfil the parasite’s life cycle makes blood-feeding a major component to vector-borne disease transmission [3].

## EXAMPLES IN PUBLIC HEALTH

Infection within both the vector and host can affect blood-feeding. Malaria is a well-studied example that highlights these dual impacts.

Mosquitoes harbouring the non-infectious parasitic stage probe less, reducing mortality risks associated with feeding. Yet when the parasite is at the infectious stage, probing and therefore probability of transmission to the host is increased [4]. Similarly, differential requirements for the blood-meal volume that triggers host-seeking inhibition exist depending on the parasite stage, either minimizing feeding risk with low required volume when non-infectious or maximizing transmission through high required volume when infectious [4].

Within humans, infection with malaria can lead to differential mosquito attraction. When hosts carry the infective parasitic stage, specific blood components and volatile compounds on the skin are altered. This changes mosquito behaviour by increasing attractiveness as compared to the same host when non-infectious [5–7]. Although host odour is directly modified by the parasite, the ultimate effect is alteration of mosquito behaviour, which subsequently increases parasite transmission to the vector.

## EVOLUTIONARY PERSPECTIVES AND IMPLICATIONS

Malaria is but one well-explored example of a vector-borne disease with parasite-induced behavioural changes. In many pathosystems, parasites have evolved over centuries with both their hosts and vectors. This gives opportunity for host, vector and parasite adaptation, effects of which could all present as manipulations and have similar impacts on disease dynamics regardless of the cause. Consideration of possible manipulations is important for a holistic understanding of vector-borne disease systems, and should be integrated into transmission models to enable accurate depiction of disease dynamics. Knowledge of the types of behavioural alterations commonly seen with parasite manipulations could help identify appropriate targets for vector-borne disease mitigation efforts [8].

## FUNDING

This material is based upon work supported by the National Science Foundation Graduate Research Fellowship Program under Grant No. DGE-1746939.
